# Endoscopic transsphenoidal surgery for non-functioning pituitary adenoma: Learning curve and surgical results in a prospective series during initial experience

**DOI:** 10.3389/fsurg.2022.959440

**Published:** 2022-08-02

**Authors:** Julien Boetto, Irina Joitescu, Isabelle Raingeard, Sam Ng, Marine Le Corre, Nicolas Lonjon, Louis Crampette, Valentin Favier

**Affiliations:** ^1^ Department of Neurosurgery, Gui de Chauliac Hospital, Montpellier University Medical Center, Montpellier, France; ^2^ IGF, Université de Montpellier, CNRS, INSERM, Montpellier, France; ^3^ Department of Endocrinology, Lapeyronie Hospital, Montpellier University Medical Center, Montpellier, France; ^4^ Department of ENT Surgery, Gui de Chauliac Hospital, Montpellier University Medical Center, Montpellier, France

**Keywords:** Pituitary, learning curve, skull base surgery, endoscopic endonasal approach, pituitary adenama

## Abstract

**Background:**

To report the initial experience of surgery for non-functioning pituitary adenoma (NFPA) from a neurosurgeon in a dedicated residency training endoscopic transsphenoidal (ETS) program, and detail the surgical and clinical outcomes during this period.

**Methods:**

A prospective series of all patients operated for NFPA, using an ETS approach, during the three first years of experience of a newly board-certified neurosurgeon was analysed. Clinical, radiological and peri-operative data were collected. Extent of resection (EOR) was determined by formal volumetric analysis. Impact of the learning curve and predictive factors of gross total resection (GTR) were determined.

**Results:**

Fifty-three patients with NFPA were included in this prospective cohort which was divided in two periods of time (“First period”: 30 first cases, and “second period”: 23 following cases). Baseline characteristics of the patients in the two periods were similar. Overall occurrence of complication was 22% and was not significantly different in the two periods of time. No patient had severe neurological complication. Gross total resection was achieved in 70% of patients. Mean Extent of resection was 96%. In a multiple linear regression model, a higher EOR was positively correlated with experience (*p* = 0.018) and negatively correlated with Knosp Score equal to 4 (*p* < 0.001). Predictive factors for GTR were Higher Knosp grade (*p* = 0,01), higher pre-operative volume (*p* = 0.03), and second period of time (*p* = 0.01).

**Conclusion:**

NFPA surgery can be safe and efficient during the learning period. Dedicated intensive learning, careful patient selection and multidisciplinary work are key to shorten the learning curve and achieve satisfactory results.

## Introduction

The endoscopic endonasal approach to the sella has been widely used since the late 1990s for pituitary adenoma surgery (1). First described by R. Jankowski in 1992 (2), endoscopic guided transsphenoidal surgery was standardized in clinical practice by Jho and Carrau (3, 4) and Capabianca (5). Though it allows similar gross total resection rates as microscopic approaches (6), the enhanced illumination and visualization of both anatomical elements and the pituitary lesion provided by the endoscope has led most pituitary surgeons to turn to purely endoscopic procedures (1). However, a key factor in the use of the endoscope is the significant learning curve associated with its safe and effective use (7, 8). It is often considered to be less intuitive than the microscope because of a potential decreased ability to use instruments under the direct vision of the operating surgeon. As a result, several studies report a steep learning curve before proficiency is achieved when converting to this challenging method. However, there are conflicting reports and no clear consensus as to the endpoint of the learning curve in order to achieve satisfactory results, with published reports varying between 15 and 100 procedures as being necessary (8–10). Indeed, recent publications show that the effect of the learning curve is still visible even after extensive experience of endoscopic surgery (11).

Studies focusing on this topic generally describe very experienced pituitary surgeons who have turned from microscopic to endoscopic surgery. The next generations of pituitary surgeons will undoubtedly commence their experience with fully endoscopic procedures after a residency period and a dedicated training period marked by the predominance of the endoscope. The possibility of transposing learning curve results from surgeons who are experienced in microscopic vision and gestures who have converted to endoscopic surgery, to surgeons who are fully trained in endoscopy is uncertain: very few studies have focused on surgical results during the initial training period in endoscopic surgery for less experienced surgeons who have no previous experience with microscopic pituitary surgery.

In this study, we report an initial experience of fully endoscopic non-functioning pituitary adenoma (NFPA) surgery from a neurosurgeon who had a dedicated endonasal endoscopic training during residency, and without any experience of previous microscopic pituitary surgery. The main objective was to assess the impact of experience on surgical results and to characterize a potential learning curve. The secondary objective was to examine predictive factors of gross total resection and surgical complications.

## Material and methods

### Patient population and study design

A prospective cohort study including all patients undergoing endoscopic endonasal surgery between November 2017 and November 2020 by a single neurosurgeon (JB) was established. At the beginning of the study, the neurosurgeon (JB) was starting his experience as a board-certified neurosurgeon. Patients who fulfilled the following criteria were included in this study: patients with non-functioning pituitary adenomas (NFPA), operated through endoscopic non-extended approach (no parasellar or transplanar resection). Patient's medical history was prospectively recorded, including demographics, tumor type, visual status, endocrine status, operative data (approach, total operative time, cerebro-spinal fluid (CSF) leak, closure), post-operative complications, need for revision surgery, quality of resection with volumetric assessment, and length of hospital stay (LOS).

All patients provided informed consent for the prospective or retrospective analysis of their clinical and radiological information. The study received the approval of the Institutional Review Board (n°2019 IRB_MTP_12-02) of the University Hospital of Montpellier.

### Radiological evaluation

All patients underwent magnetic resonance imaging (MRI) with 1.5-Tesla T2-weighted and T1-weighted imaging, with and without gadolinium enhancement. Pituitary adenomas were classified according to the maximum tumor diameter into microadenomas (<10 mm), macroadenomas (between 10 and 40 mm), and giant adenomas (>40 mm). Tumor extension was radiologically defined in the prospective group using the modified Knosp Score (12). “Invasion” represents a composite criterion based on radiological findings (cavernous sinus or sphenoidal sinus invasion), intraoperative examination and histopathological findings, as defined by the Hypopronos score (13).

Patients underwent a systematic postoperative CT-scan in the first 24 h following surgery and a 6-month follow-up MRI. A volumetric assessment was performed using HOROS software (Nimble Co LLC d/b/a Purview in Annapolis, MD, USA) based on presurgical MRI and 6-month post-operative MRI.

### Ophthalmologic evaluation

All patients underwent pre-operative and post-operative ophthalmological examinations at 3 months and 12 months follow-up. Visual acuity and Goldman visual field were tested for both eyes. Ophthalmologic results were divided into four groups: worsened when visual acuity or visual field decreased; unchanged when no post-operative change occurred; partial recovery; and complete recovery (visual acuity and visual field both returned to normal).

### Hormonal assessments

All patients underwent pre-operative static endocrinological examination including measurement of serum levels of prolactin, luteinizing hormone, follicle-stimulating hormone, testosterone (in men) and estradiol (in women), 8:00 am adrenocorticotropic hormone and cortisol, insulin-like growth factor-I, thyroid stimulating hormone and free T3 and T4. These measurements were repeated at 1 month, 3 months and 1 year after surgery. The results were classified as “worsened” when at least one new pituitary deficit occurred, as “partial recovery” when at least one pituitary deficit recovered, as “complete recovery” when all pituitary deficits recovered, and as “unchanged” when no change occurred. Post-operative diabetes insipidus (DI) was defined as daily diuresis over 3L with urinary osmolarity <1005 mOsmol/kg. Endocrine deficits were considered as permanent when found to be persistent at 1 year after surgery.

### Operative technique

Procedures were performed with a 30° endoscope, using a unilateral right nostril transseptal fully endoscopic approach, as described previously (14). The nasal phase was generally performed by an ENT, and the sellar phase was performed systematically by the neurosurgeon. Neuronavigation was used in selected cases (particularly in surgery for recurrence). A large anterior sphenoidotomy was performed. A bone resection of the sellar floor was performed with conservation of the inferior margin to facilitate closure at the end of the procedure. The sellar dura was incised and the tumor was removed according to classical microsurgery methods, using cottonoids, curettes, microspatula and suction devices. The chopsticks technique (15) was generally used and no endoscope-holder was used. A Vasalva manoeuvre was generally performed at the end of the resection to confirm the absence of CSF leak or in case of insufficient prolapse of the suprasellar extension of the lesion. After resection, haemostasis was achieved using warm saline irrigation and compression with cottonoids. A solution of thrombin (SURGIFLO Thombin, Ethicon, Somerville, New jersey, USA) was generally used to secure the intrasellar hemostasis. In case of CSF leak, an abdominal fat graft was used with a fibrinogen sealant (Tisseel, Baxter, Deerfield, Illinois, USA) to fill the sella. No cases required the use of a naso-septal flap.

### Post-operative complications

Post-operative complications were recorded and categorized as follows: surgical complications (CSF leak, meningitis, sino-nasal complications, neurological complications, visual impairment), endocrine complications (permanent anterior pituitary deficiency, permanent diabetes insipidus), and medical complications (venous thrombosis, infection …). Evaluation of sino-nasal morbidity was done by in-office endoscopy examination, performed by an ENT physician 10 to 15 days after the surgery.

### Extent of resection

The extent of resection (EOR) was evaluated on the basis of the pre- and 6 months post-operative MRI with volumetric assessment. EOR was judged as gross total resection (GTR) when no residual tumor was present. When the residual tumor was calculated to be less than 10%, the resection was judged as subtotal resection (STR). Partial resection (PR) was defined as residual tumor that was greater than 10%. Partial and subtotal resection were grouped as “incomplete resection”.

### Learning curve assessment

To assess the impact of the learning curve, two strategies were used. First, patients were numbered in a chronologic fashion and correlations were established with the increasing number of cases. Second, the cohort was arbitrarily divided into a “first period” (first 30 patients, corresponding to the two first years of experience) and a “second period” (23 following patients, corresponding to the last year of experience).

### Statistical analysis

Categorical variables were compared using Fisher's exact test or Chi-Square test, while continuous variables were compared using either a paired Student t-test or the Mann-Whitney test, depending on normal distribution of the data, or one-way ANOVA when more than two groups were compared. Statistical analysis performed in a sequential fashion delineated a learning curve for operative time, length of hospital stays, EOR or tumor residual volume using Pearson correlations (with r defined as the correlation coefficient). To study factors correlated with extent of resection, a multiple linear regression model was build including all factors potentially affecting the quality of resection (based on univariate correlation tests). For all statistical analyses, tests were two-sided and *p* < 0.05 was considered statistically significant. Statistical tests were performed using RStudio 1.3.1093 software.

## Results

### Participants

Among the 86 patients operated for skull base lesions through ETS during the prospective inclusion period (2017–2020), 53 patients had NFPA and were included in the present study.

### Demographic and radiological assessment

Main baseline demographics and radiological characteristics of the cohort are summarized in Table 1. As expected for NFPA, no patient had microadenoma. 45% of lesions were “invasive” as defined in the Hypopronos Score. Mean pre-operative volume was 7.37 ± 7 cm^3^. 19% of cases were recurrence surgeries. There was no significant difference between the population or the radiological appearance (size, invasiveness) between the two periods of time (Supplementary Table S1).

**Table 1:** Demographics and tumor type.

Demographics	General cohort (*N* = 53)
Sex	
Female, *n* (%)	23 (43)
Male, *n* (%)	30 (57)
Mean age (SD)	59 (15.1)
Symptoms at diagnosis	
Mass effect, *n* (%)	34 (64)
Endocrine, *n* (%)	10 (19)
Apoplexy, *n* (%)	2 (4)
Incidental, *n* (%)	7 (13)
MRI characteristics	
Macroadenoma, *n* (%)	46 (87)
Giant adenoma, *n* (%)	7 (13)
Mean maximum diameter (mm) (SD)	27.5 (10.8)
Mean Preoperative Volume (cm^3^) (SD)	7.37 (7.03)
Invasion, *n* (%)	24 (45)
Knosp Score, *n* (%)	
0	5 (9.4)
1	12 (23)
2	16 (30)
3a	8 (15)
3b	3 (5.7)
4	9 (17)
Procedure	
First surgery	43 (81)
Recurrence surgery	10 (19)

### Operative course and post-operative complications

Results concerning the operative course and post-operative complications are summarized in Table 2. Mean operative time was 121 min. No patient died from a complication of the surgery, had an internal carotid artery injury, a post-operative neurologic deficit, or a visual deterioration. Overall, the occurrence of complications was 22%: 5 patients (9%) had a surgical complication and 7 patients (13%) an endocrine complication (defined as a permanent diabetus insipidus or a worsened hypopituitarism). 3 patients needed a revision surgery (one for a hematoma of the sella and two for a post-operative CSF leakage). Occurrence of complication depending on the period of time are summarized in Supplementary Table 2.

**Table 2:** Intra-operative course and post-operative complications.

	General Cohort (*N* = 53)
Mean Total Operative time^a^, min (SD)	121 (29)
Mean length of stay, days (SD)	7.6 (2.3)
Quality of resection	
Complete resection, *n* (%)	37 (70)
Incomplete resection, *n* (%)	16 (30)
STR, *n* (%)	8 (15)
PR, *n* (%)	8 (15)
Mean extent of resection (%), (SD)	96 (8)
Visual outcome	
Complete recovery, *n* (%)	9 (28)
Partial recovery, *n* (%)	9 (30)
Stabilization, *n* (%)	10 (31)
Worsening, *n* (%)	0 (0)
Surgical complication	
Meningitis, *n* (%)	1 (1.8)
Hematoma of the sella, *n* (%)	1 (3)
Permanent CSF leak, *n* (%)	2 (3.7)
Internal carotid injury, *n* (%)	0 (0)
Neurological deficit, *n* (%)	0 (0)
Nasal complication	
Epistaxis, *n* (%)	0 (0)
Anosmia, *n* (%)	1 (1.8)
Endocrine complication	
Permanent DI, *n* (%)	2 (3.7)
Worsened hypopituitarism, *n* (%)	5 (9.4)
Need for reintervention, *n* (%)	3 (5.6)
Death, *n* (%)	0 (0)

^a^ Total operative time includes both nasal and sellar phase.

### Extent of resection

**Figure 1:**
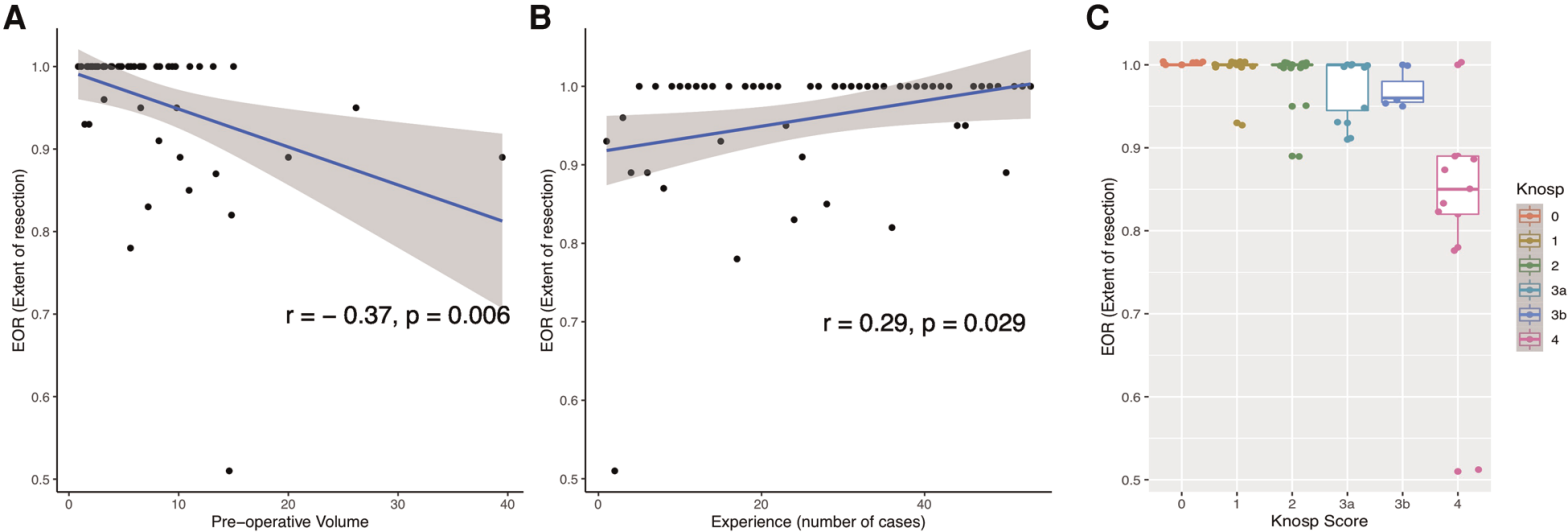
Representation of factors correlated with EOR (Extent of resection). (**A**) Scatterplot showing the correlation of EOR with pre-operative volume. (**B**) Scatterplot showing the correlation of surgical experience with EOR. (**C**) Boxplot showing the EOR depending on Knosp Scores.

**Figure 2:**
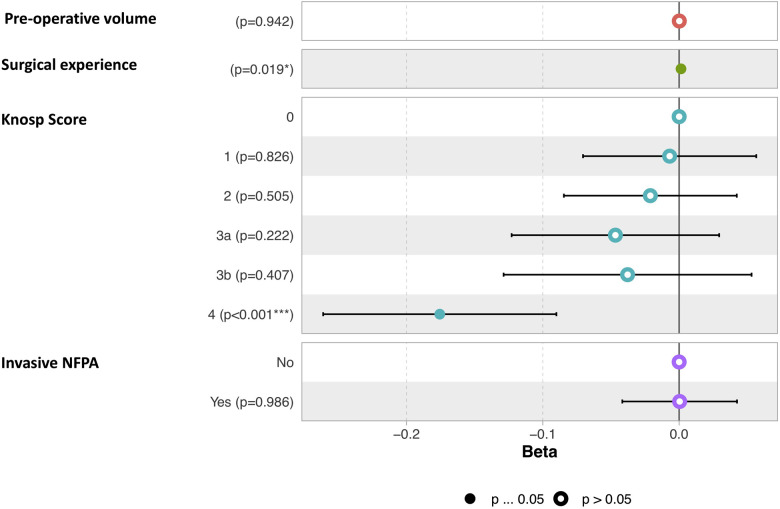
Graphic representation of the multiple linear regression model of factors correlated to EOR. The term “invasive” refers to the Hypopronos classification.

The mean pre-operative volume was 6.65 ± 7 cm^3^ (Range 1.2—39.5 cm^3^) and the mean residual tumor volume was 0.62 ± 1.4 cm^3^ (Range 0–7.22 cm^3^). The mean extent of resection (EOR) was 96%. GTR rate was 70%, STR rate was 15% and PR rate was 15% (Table 2). A higher EOR was positively correlated with a lower pre-operative volume (*r* = −0.37, *p* = 0.006), increasing surgical experience (*r* = 0.29, *p* = 0.029), and was associated with a lower invasiveness (Mann-Whitney Test, *p* = 0.003) and a lower Knosp Score (One-way ANOVA, *p* = 5.69e-07). Results are illustrated Figure 1. In a multiple linear regression model, Knosp grade 4 (*p* < 0.001) and starting the surgical experience (*p* = 0.019) were independently correlated with a lower EOR (Figure 2).

### Predictive factors of GTR

Results of the univariate analysis of factors associated with a GTR are summarized in Table 3. Incomplete resection was associated with higher tumor volumes**,** invasiveness and a higher Knosp Score. Among NFPA patients with Knosp Scores of 0, 1 or 2, the GTR rate was 90%, whereas GTR rate was 35% in those patients harbouring NFPA with a Knosp score of 3 or more. Recurrence surgery had no clear impact on the rate of GTR.

**Table 3:** Factors associated with GTR during initial experience.

	Incomplete resection (*n* = 16)	Complete resection (*n* = 37)	*p*-value
Demographics			
Mean Age	67 (14)	57 (13)	0.021^b^
Sex (female)	7 (44)	16 (43)	1^c^
MRI Characteristics			
Mean Preoperative Volume (cm^3^) (SD)	12.1 (9.8)	5.3 (3.6)	0.004^b^
Invasion, *n* (%)	12 (74)	12 (32.4)	0.004^a^
Knosp Score, *n* (%)			
0	0 (0)	5 (14)	<0.001^c^
1	1 (6.2)	11 (30)	
2	2 (12)	14 (38)	
3a	3 (19)	5 (14)	
3b	2 (12)	1 (2.7)	
4	8 (50)	1 (2.7)	
Procedure			
First surgery, *n* (%)	13 (81)	30 (81)	1^c^
Recurrence surgery, *n* (%)	3 (19)	7 (19)	
Learning curve			
First period (*n* = 30), *n* (%)	12 (75)	18 (49)	0.07^a^
Second period (*n* = 23), *n* (%)	4 (25)	19 (51)	

^a^ *χ*^2^ test.

^b^ Mann-Whitney test.

^c^ Fisher's exact test.

### Effect of the learning curve

**Figure 3:**
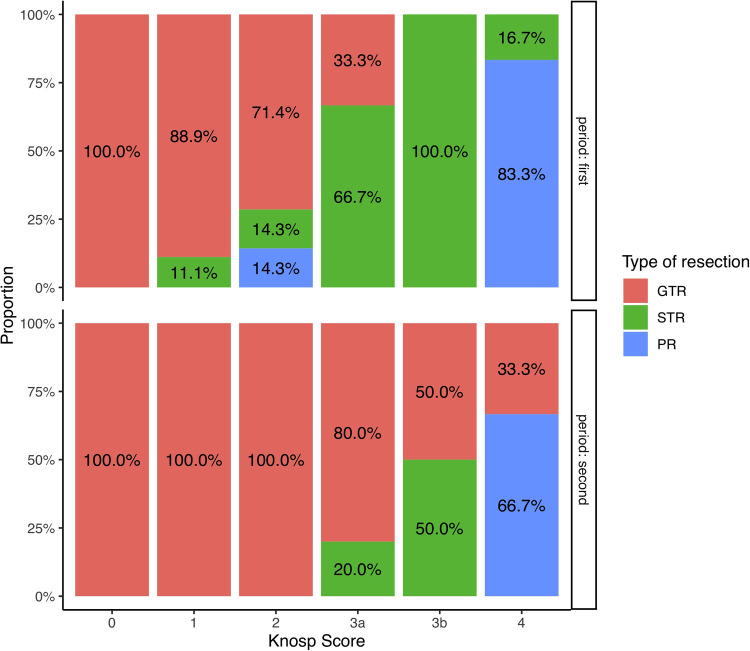
Bar-plot representation of the resection rates depending on the Knosp score during the first and the second period of time.

Increasing surgical experience was correlated with a higher EOR (*r* = 0.29, *p* = 0.029), and a diminution of the operating time and the LOS (r = −0.31, *p* = 0.021 and r = −0.25, *p* = 0.02, respectively). The rates of resection depending on Knosp grade and Period of time are illustrated Figure 3. The occurrence of complication was not correlated with the period of time (Supplementary Table S2). All patients who needed a revision surgery were operated during the second period of time (Supplementary Table S2).

## Discussion

In this prospective cohort of patients operated on using an endoscopic endonasal approach for a NFPA during the three first years of experience, we report that (1) these procedures can be performed with relatively low complication rates and satisfactory results during the learning period; (2) the effect of the learning curve was clearly visible on the EOR but not on the complication rate; (3) Starting the surgical experience and a Knosp score equal to 4 were the main independent factors correlated to lower EOR.

As in other specialities, a clear relationship between the level of the surgeon's experience and outcomes has been demonstrated in pituitary surgery, often described as the “learning curve” (16). It is supposed that patients treated by more experienced surgeons experience fewer complications, have a lower mortality rate, and lower hospital charges. Based on the assumption that technical performance improves with experience, learning curve analyses in surgical specialties have aimed to define the number of cases required to perform a certain procedure until outcomes reach an average rate or plateau. While cumulative sum analyses have been sometimes used to assess the improvement in surgical technical skills, they are not easy to construct in the case of complex procedures like tumoral resections, where an extremely complex array of variables is involved in intra-operative decision-making, and binary criteria of surgical “success” versus “failure” are not possible to identify (17, 18).

The endoscopic transsphenoidal approach is widely performed in pituitary surgery nowadays, with increasing evidence supporting its use for the gradual replacement of microsurgical techniques (6). The transition between these two techniques is the subject of several studies which aimed to define the learning curve (7, 8, 10, 19). Rates of GTR, the rate of hormonal remission (for hormone secreting adenomas) and operative times are generally markers of surgical efficiency, while complication rates are markers of surgical safety/skills. Among the latter, post-operative CSF leak is the most frequent complication and is generally defined as a pertinent marker reflecting the learning curve. Nevertheless, results from the literature are contradictory, with the required number of procedures estimated to reach a plateau ranging from 15 to more than 100, depending on the endpoint selected. In a recent study, Shikary *et al* reported a number of 120 procedures required for a team to reach a plateau of 125 min for mean operative length, and 100 procedures to stabilize CSF leak rates below 5% (10). However, the authors acknowledged that the pattern of the learning curve was difficult to analyse due to multiple factors affecting the quality of the surgery (e.g. size of the lesion, expansion of exposure, reconstruction technique, patients' morphological characteristics, aggressiveness of surgery…). Other studies have emphasised that the pattern of the learning curve is not so simple: a potential “rebound effect” with a “second learning curve effect” is defined as an increase in complication rates explained by the surgeon adopting a more aggressive resection approach after becoming more comfortable performing the procedure, or selecting more challenging cases (19). On the other hand, recent studies showed that contrary to popular belief, the surgical learning curve does not plateau but continues for several years after hundreds of cases (11, 20).

The introduction of endoscopy provided a new tool for these procedures which carries its own learning curve, explaining why the vast majority of studies have been dedicated to the learning curve for a transition between microscopic and endoscopic techniques for experienced operators (8–10, 19, 21–23). However, new generations of pituitary surgeons will undoubtedly commence their experience using fully endoscopic procedures, after a residency period and dedicated training period marked by the predominance of the endoscope. Whether it is possible to translate learning curve results from surgeons experienced in the microscopic technique who retrain in endoscopy, to surgeons solely trained in endoscopy is uncertain. A recent study comparing surgical results of two surgeons raised the provocative question of whether certain advantages of endoscopic surgery may help a less-experienced surgeon to achieve outcomes similar to those of more experienced surgeons for non-functioning adenomas (24). However, the junior neurosurgeon in this study already had experience of more than 100 cases, illustrating that no study in the literature has focused on surgical results during the actual beginning of the surgical experience.

In order to address this question, we studied the early surgical results of a newly board-certified neurosurgeon who undertook a dedicated training program during his residency. This training program consisted of obtaining a comprehensive knowledge of skull base anatomy, a dedicated learning of surgical gestures through weekly cadaveric dissections in the laboratory, participation in a specific course (360 degree skull base course (4 days), IRCAD, Strasbourg, France), a clinical fellowship in a high caseload volume reference center for endoscopic endonasal surgery, and active participation in pituitary surgeries involving a multidisciplinary team including ENT surgeons during his residency.

Our results suggest that the classical drawbacks of endoscopy at the beginning of the surgical experience (e.g. lack of adaptation to endoscopic visualisation, narrow operational space, bleeding or damage to the mucosa caused by movements of the endoscope or instruments) may have been overcome by this dedicated training and the benefits obtained from a multidisciplinary surgical team, leading to a shortening of the learning curve in terms of operative time and occurrence of complications Our mean operative time was 121 min and decreased with surgical experience. It was quite similar to operative times published by other groups (10, 21, 22, 25). Besides, there was no clear effect of the learning curve demonstrated in terms of occurrence of complications : our complication rate was quite similar to those described in the literature from more experienced operators (25–28). Although intraoperative CSF leak was frequent in our series (37%), a meticulous closing strategy enabled the post-operative CSF leak rate to be limited to less than 5%, corresponding to that seen in the more advanced period of experience reported in other series (10, 21, 23). Patients who needed revision surgery for surgical complications were actually operated during the second period of this study. This suggests that the increasing of confidence in order to achieve more extensive resection (Figure 3) led to more complication during this second period of time, classically described as “the rebound effect”. In addition, no patient showed severe surgery-related complications such as carotid artery injury or neurological deficit, confirming that this procedure can be performed with an acceptable degree of safety during an initial experience course, with the only condition being an intense dedicated training program and a multidisciplinary working environment.

Results on the extent of resection showed 70% of GTR, and a mean EOR of 96%. Though EOR results could be improved in comparison to those published by very experienced teams (1, 6, 28), we demonstrated a clear impact of the learning curve that led to acceptable results during the second period of time (Figure 3): every patient having NFPA with Knosp score <3 had GTR after the 30 first patients operated and the mean volume of residual tumor was 0.6 cm^3^ for the whole cohort. Main Predictive factors of higher EOR were the Knosp Score and the surgical experience. The invasiveness, as defined by the Hypopronos Score was also associated with decreased rates of GTR. These results are similar to those that have been published by expert teams. (6, 24, 29, 30).

Our results also confirm that NFPA are ideal cases for initial experience in pituitary surgery. While GTR is not always the goal of surgery in NFPA, surgical resection of hormone secreting adenomas is often a more complex procedure associated with higher complication rates, as well as having a strict necessity of gross total resection. As recommended by expert teams and master surgeons in this field, we chose to focus our initial experience on NFPA before attempting surgical resection of hormone secreting adenomas. Though our results could largely be improved, we demonstrated that in the era of fully endoscopic procedures, an intensive dedicated training program enables surgical resection of NFPA to be performed with an acceptable safety/efficiency profile from the very early experience period. We describe the complication rates and EOR during this early period using a strong methodology, and suggest that the use of the endoscope starting at the very beginning of the experience period may be associated with a shortening of the learning curve. These findings may have useful implications for surgical education, future studies on the learning curve in pituitary surgery and development of patient safety measures in the early surgical experience period.

### Limitations

Firstly, the prospective cohort in our study is relatively small (53 patients), and a more extensive series would be necessary to better assess the impact of the learning curve. Secondly, our series concerned only NFPA and we believe that our conclusions cannot be translated to surgery for other types of pituitary lesions.

## Conclusion

The acquisition of expertise in endoscopic endonasal surgery requires engaging in a dedicated training program. Provided that the prerequisite skills (perfect knowledge of nasal cavities and anatomy of the sellar region learned from dissection practice in the laboratory, endoscopic manipulation techniques, understanding of preoperative imaging) have been appropriately mastered, resection of NFPA carries an acceptable complication rate and produces satisfactory outcomes even during the very early experience period of the surgeon. Meticulous patient selection and a multidisciplinary work environment are also key in achieving good results.

## Data Availability

The raw data supporting the conclusions of this article will be made available by the authors, without undue reservation.
