# Supplementary material for: Endoscopic transsphenoidal surgery for non-functioning pituitary adenoma: Learning curve and surgical results in a prospective series during initial experience
Source: Front Surg. 2022 Aug 2;9:959440. doi: 10.3389/fsurg.2022.959440 (PMC9379140; doi:10.3389/fsurg.2022.959440)
Supplement: Supplementary file 1 [file Table_1.docx]

Supplementary Table 1: Demographics and tumor type by period

| Demographics | General Cohort (N=53) | First Period (N=30) | Second Period (N=23) | P-value |
| --- | --- | --- | --- | --- |
| **Sex** |  |  |  |  |
| Female, n (%) | 23 (43) | 12 (40) | 11 (48) | 0.6 ^a^ |
| Male, n (%) | 30 (57) | 18 (60) | 12 (52) |  |
| **Mean age** (SD) | 59 (15.1) | 62 (11.6) | 58 (12.4) | 0.4 ^b^ |
| **Symptoms at diagnosis** |  |  |  |  |
| Mass effect, n (%) | 34 (64) | 20 (66) | 14 (61) | 0.9 ^a^ |
| Endocrine, n (%) | 10 (19) | 5 (17) | 5 (22) |  |
| Apoplexy, n (%) | 2 (4) | 1 (3.3) | 1 (4.3) |  |
| Incidental, n (%) | 7 (13) | 4 (13) | 3 (13) |  |
| **MRI characteristics** |  |  |  |  |
| Macroadenoma, n (%) | 46 (87) | 27 (90) | 19 (82) | 0.69 ^a^ |
| Giant adenoma, n (%) | 7 (13) | 3 (10) | 4 (18) |  |
| Mean maximum diameter (mm) (SD) | 27.5 (10.8) | 26.8 (7.7) | 27.8 (12) | 0.21^b^ |
| Mean Preoperative Volume (cm^3^) (SD) | 7.37 (7.03) | 6.57 (7.19) | 8.41 (6.31) | 0.33^b^ |
| Invasion, n (%) | 24 (45) | 13 (43) | 11 (47) | 0.3 ^a^ |
| Knosp Score, n (%) |  |  |  | 0.3 ^a^ |
| 0 | 5 (9.4) | 4 (13) | 1 (4.3) |  |
| 1 | 12 (23) | 9 (30) | 3 (13) |  |
| 2 | 16 (30) | 7 (23) | 0 (39%) |  |
| 3a | 8 (15) | 3 (10) | 5 (22%) |  |
| 3b | 3 (5.7) | 1 (3.3) | 2 (8.7) |  |
| 4 | 9 (17) | 6 (20) | 3 (13) |  |
| **Procedure** |  |  |  |  |
| First surgery | 43 (81) | 23 (77) | 20 (87) | 0.5 ^a^ |
| Recurrence surgery | 10 (19) | 7 (23) | 3 (13) |  |

a: Chi 2 test; b: Mann-Whitney test; c: Fisher’s exact test (comparison between the two periods of time).
