# Supplementary material for: Endoscopic transsphenoidal surgery for non-functioning pituitary adenoma: Learning curve and surgical results in a prospective series during initial experience
Source: Front Surg. 2022 Aug 2;9:959440. doi: 10.3389/fsurg.2022.959440 (PMC9379140; doi:10.3389/fsurg.2022.959440)
Supplement: Supplementary file 2 [file Table_2.docx]

Supplementary Table 2: Intra-operative course and post-operative complications by period

|  | General Cohort (N=53) | First Period (N=30) | Second Period (N=23) | P-value |
| --- | --- | --- | --- | --- |
| **Mean Operative time, min (SD)** | 121 (29) | 127 (32) | 113 (22) | 0.08^b^ |
| **Mean length of stay, days (SD)** | 7.6 (2.3) | 8 (2.7) | 7 (1.5) | 0.12 ^b^ |
| **Quality of resection** |  |  |  |  |
| Complete resection, n (%) | 37 (70) | 18 (60) | 19 (83) | 0.14^a^ |
| Incomplete resection, n (%) | 16 (30) | 12 (40) | 4 (17) |  |
| STR, n (%) | 8 (15) | 6 (20) | 2 (8.7) |  |
| PR, n (%) | 8 (15) | 6 (20) | 2 (8.7) |  |
| **Mean extent of resection (%), (SD)** | 96 (8) | 94 (10) | 98 (4) | 0.08 |
| **Visual outcome** |  |  |  |  |
| Complete recovery, n (%) | 9 (28) | 8 (27) | 10 (43) | 0.3 |
| Partial recovery, n (%) | 9 (30) | 9 (30) | 3 (13) |  |
| Stabilization, n (%) | 10 (31) | 13 (43) | 10 (43) |  |
| Worsening, n (%) | 0 (0) | 0 (0) | 0 (0) |  |
| **Surgical complication** |  |  |  |  |
| Meningitis, n (%) | 1 (1.8) | 1 (3.3) | 0 (0) | NS |
| Hematoma of the sella, n (%) | 1 (3) | 0 (0) | 1 (4.3) | NS |
| Permanent CSF leak, n (%) | 2 (3.7) | 0 (0) | 2 (8.6) | NS |
| Internal carotid injury, n (%) | 0 (0) | 0 (0) | 0 (0) | NS |
| Neurological deficit, n (%) | 0 (0) | 0 (0) | 0 (0) | NS |
| **Nasal complication** |  |  |  |  |
| Epistaxis, n (%) | 0 (0) | 0 (0) | 0 (0) | NS |
| Anosmia, n (%) | 1 (1.8) | 1 (3.3) | 0 (0) | NS |
| **Endocrine complication** |  |  |  |  |
| Permanent DI, n (%) | 2 (3.7) | 1 (3.3) | 1 (4.3) | NS |
| Worsened hypopituitarism, n (%) | 5 (9.4) | 4 (13) | 1 (4.3) | .12^c^ |
| **Need for reintervention, n (%)** | 3 (5.6) | 0 (0) | 3 (13) | NS |
| **Death, n (%)** | 0 (0) | 0 (0) | 0 (0) | NS |

a: Chi 2 test; b: Mann-Whitney test; c: Fisher’s exact test. NS: not significant. (comparison between the two periods of time).
